# Remission of an HHV8-related extracavitary primary effusion lymphoma in an HIV-positive patient during antiretroviral treatment containing dolutegravir

**DOI:** 10.1186/s12981-019-0230-6

**Published:** 2019-07-27

**Authors:** Laura Campogiani, Carlotta Cerva, Gaetano Maffongelli, Elisabetta Teti, Livio Pupo, Sara Vaccarini, Maria Cantonetti, Alfredo Pennica, Massimo Andreoni, Loredana Sarmati

**Affiliations:** 1 Clinical Infectious Disease, Department of System Medicine, Tor Vergata University, Rome, Italy; 2 Department of Hematology, Tor Vergata University, Rome, Italy; 3 Clinical Infectious Disease, Sant´Andrea Hospital, Rome, Italy; 4 Clinical Infectious Diseases, Belcolle Hospital, Viterbo, Italy

**Keywords:** HHV8, Primary effusion lymphoma, Dolutegravir

## Abstract

**Background:**

Human herpes virus 8 (HHV8) is the causative agent of Kaposi’s sarcoma and has been associated with an increasing number of hematologic diseases such as primary effusion lymphoma (PEL) (both classic and extracavitary form), multicentric Castleman disease and the germinotropic lymphoproliferative disorder. PEL is a rare B cell non-Hodgkin lymphoma that primarily affects immunocompromised patients; aggressive chemotherapy and antiretroviral therapy (ART) with protease inhibitors have been used, with poor results. We present a case of extracavitary PEL in an HIV-infected patient, regressed after ART initiation.

**Case presentation:**

A 42-year-old male was admitted to the emergency room because of several months of malaise, fever and progressive deterioration of the general conditions. On physical examination soft non-painful subcutaneous masses were palpable at retronuchal, retroauricolar and thoracic regions. HIV serology resulted positive: HIV plasma viremia was 782,270 copies/mL, CD4 103 cells/mL. The excision of one of the masses, metabolically active at a positron emission tomography (PET-CT) scan, revealed an HHV8-related extracavitary PEL. HHV8 plasma viremia was 44,826 copies/mL. ART with tenofovir alafenamide/emtricitabine/dolutegravir was started together with ganciclovir for cytomegalovirus chorioretinitis. The progressive disappearance of the masses was seen after 6 weeks of ART, and a PET-CT scan resulted completely negative at 3 months. After 19 months of ART the patient was in remission of PEL, HIV viremia was undetectable (< 20 copies/mL), CD4 count was 766 cells/mL and HHV8 viremia was undetectable.

**Conclusions:**

In this clinical case, the complete regression of PEL has been achieved after the immune recovery, as a consequence of ART introduction, without chemotherapy. It cannot be excluded that ganciclovir, used for the treatment of CMV chorioretinitis, may have contributed to the control of HHV8 replication. Whether to try or not a conservative approach in HIV-infected PEL patients must be carefully evaluated, considering the patient’s characteristics and the prognostic factors.

## Background

Primary effusion lymphoma (PEL) is a rare B cell non-Hodgkin lymphoma (NHL) that accounts for 0.3% of all NHL in the general population. Men are more commonly affected than women, with a male to female ratio of 6:1 [1]. PEL is typically associated with impairment of immunity, and it affects the elderly, often in HHV8 endemic areas, transplanted populations, and above all, HIV-infected patients with a low CD4 count. In this population PEL can reach up to 4% of all AIDS related NHL [2]. PEL has been associated with human herpes virus 8 (HHV8, also called KSHV, Kaposi sarcoma-associated herpes virus), a gamma herpesvirus which was discovered in 1994 inside the Kaposi’s sarcoma (KS) cells of an HIV-infected patient. Since then, HHV8 has been associated with a wide variety of lymphoproliferative disorders such as PEL and multicentric Castleman disease (MCD) [3–6]. In the new lymphoma classification, other pathological entities have been correlated with HHV8 infection, broadening the spectra of its clinical manifestation [7].

PEL itself might present in different forms: the classic presentation, involving serous cavities with relapsing malignant effusion in the absence of a tumour mass, and the extranodal form that presents with masses in different organs mainly in lymph nodes, gastrointestinal tract, central nervous system and skin, either with or without the presence of effusions. In spite of intensive chemotherapy regimens, a poor overall survival has been reported both in the classic and extranodal forms, hardly overcoming 6 months after diagnosis A better disease-free survival has been reported for extracavitary PEL [1, 2, 8]. Given the rarity of the condition, no standard treatment has been identified, with a wide variety of treatment approaches described in the literature and different chemo regimens have been reported [8–24].

Here, we present a case of extranodal PEL in an antiretroviral treatment naïve patient, which spontaneously regressed after the initiation of ART.

## Case presentation

A 42-year-old Italian male was admitted at the emergency room complaining of several months of malaise, fever and progressive deterioration of general condition. He referred no significant past medical history. On admission, he was found to be febrile (38.7 °C) and tachycardic (110 bpm) with no respiratory complaints. Laboratory tests showed a severe anaemia (Hb 7 g/dL), lymphopenia (WBC 2960 cells/mL), and a slight c-reactive protein (CRP) alteration of 15.48 mg/L. Physical examination was unremarkable, except for palpable lymph nodes at the inguinal and axillary stations, and palpable soft non-painful masses at retronuchal, retroauricular and thoracic regions. A serological test for HIV was positive; the HIV plasma viremia was 782,270 copies/mL and the CD4 count was 103 cells/mL (16%, CD4/CD8 ratio 0.26). Heterosexual relationships were reported as possible factor for HIV infection acquisition.

The patient was promptly started on ART with dolutegravir (DTG) and tenofovir alafenamide/emtricitabine (TAF/FTC), with no adverse effects. HIV staging showed the presence of cytomegalovirus (CMV) chorioretinitis, for which ganciclovir 360 mg twice daily (5 mg/kg/day every 12 h) was started, and the presence of HHV8, detected using polymerase chain reaction (44,826 copies/mL) with no other coinfections—Table 1.

**Table 1:** Serological and virological screening of the patient

	Antibody screening		Viremia (copies/mL)
Herpes simplex 1/2	HSV1 IgG positive HSV2 IgG negative	IgM negative	NA
EBV	IgG VCA positive IgG EBNA positive IgG EA negative	IgM negative	< 55
Varicella zoster virus	IgG positive	IgM negative	NA
CMV	IgG positive	IgM negative	175
HHV8	IgG positive		44,826
HCV	Negative		NA
HBV	HBsAb positive		Negative
HAV	IgG negative	IgM negative	NA
Syphilis	Negative		NA
Tuberculosis	Mantoux negative	TB gold negative	NA
Toxoplasmosis	IgG negative	IgM negative	NA

*NA* not applicable

**Fig. 1:**
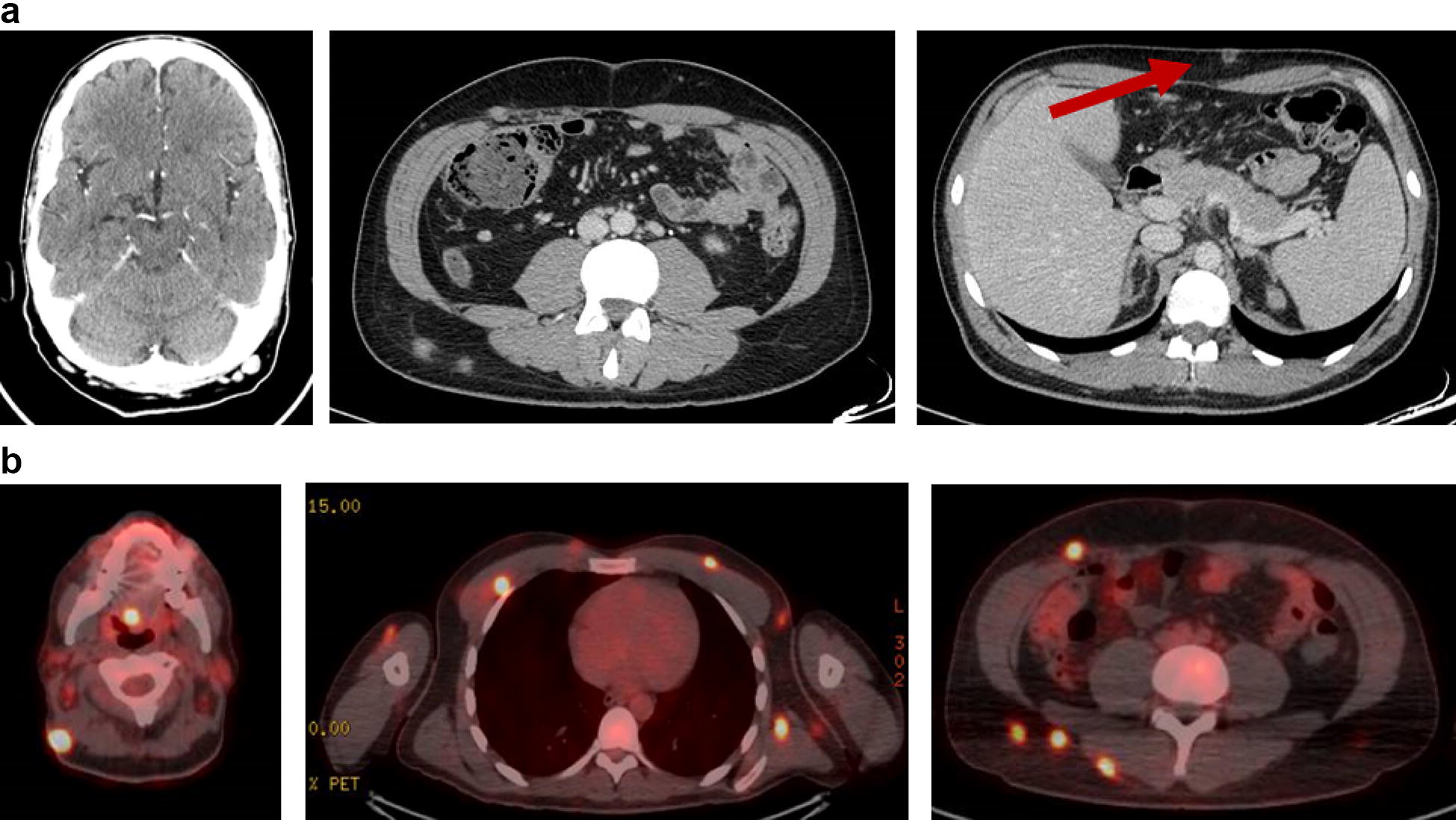
Radiologic exams. **a** Total body CT scan. Several subcutaneous solid masses with irregular shape and contrast enhancement. (arrow on the excised lesion). **b** PET-CT total body scan. Ubiquitous hyperfixating lesions, distributed both over and under the diaphragm

**Fig. 2:**
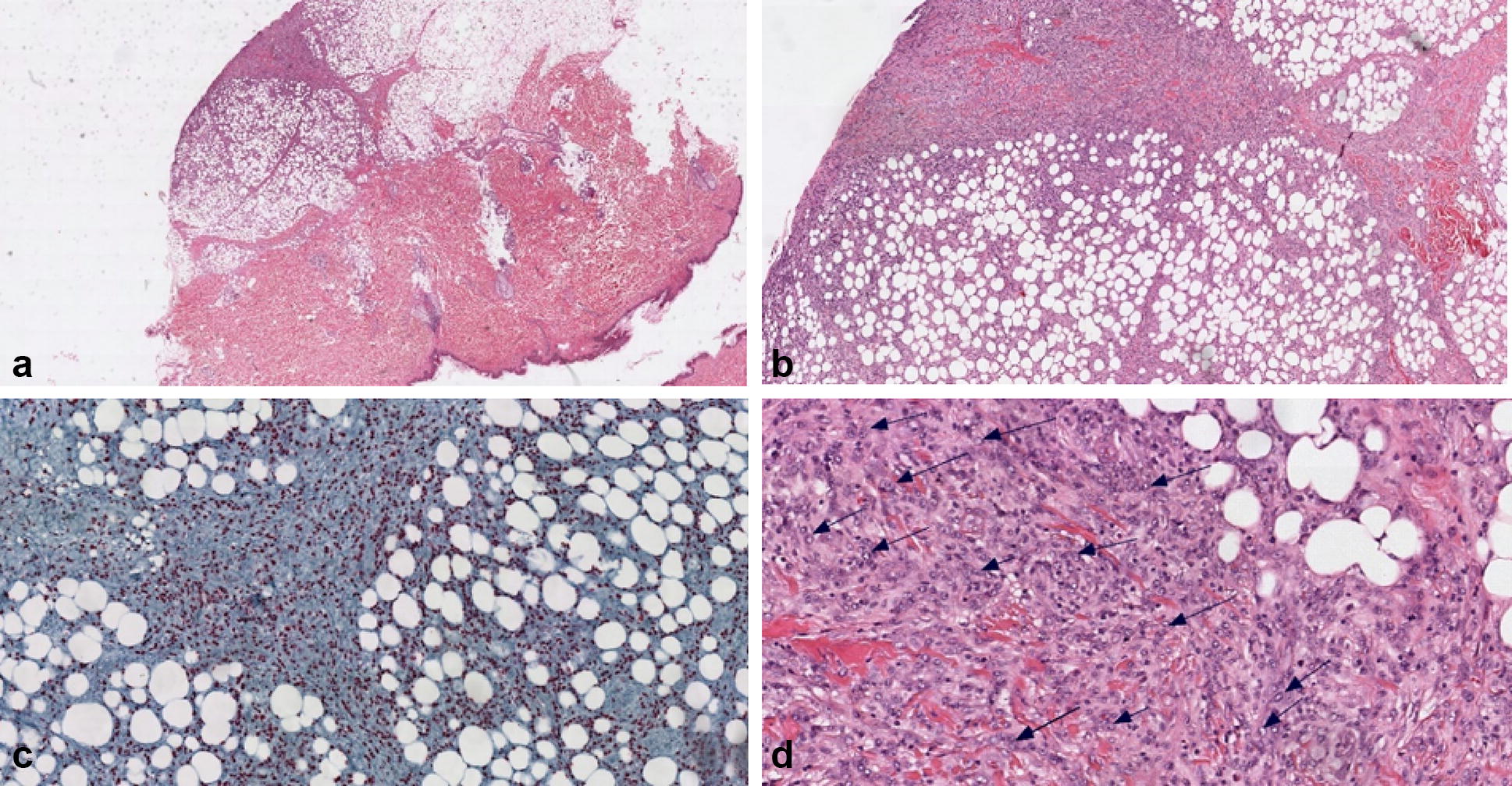
Anatomo-pathology and immunohistochemistry. LCA±; CD43±; CD30+  MUM-1+; IRF4+; CD5−; CD2−; CD3−; CD4−; CD8−; HHV8+; podoplanin−; CD34−; CD31−; S100−; CD138−; CD79 alpha−; CD20−; CD68±; CD56−; CD15−; MPO focal positivity; EBNA negative. **a** Haematoxylin-eosin (H&E) ×1; epithelial cells and adipocytes are visible in the excised formation. **b** H&E deep periferic portion, ×4 magnification. **c** HHV8 presence, detected using anti latent nuclear antigen (LANA-1) antibodies, evidenced in red, ×4 magnification. **d** Black arrows pointing at atypical cells, with irregular nuclei and numerous nucleoli, ×20 magnification

Due to a persistent fever of unknown origin, after 2 weeks of effective anti-CMV and ART, the patient was submitted to a total body CT scan. Several subcutaneous solid masses with irregular shape and contrast enhancement were seen distributed both above and under the diaphragm. A positron emission tomography (PET-CT) scan revealed these masses to have a high metabolic activity, with hyperfixation of bone lesions in the skull, both femurs and tibiae; a weaker metabolic activity was also present in the major lymphatic stations—Fig. 1. The excision of one of the metabolically active masses revealed an HHV8-related high grade lymphoproliferative tumour. HHV8 presence was detected with anti latent nuclear antigens (LANA-1) antibodies. The immunohistochemistry results are as follows: LCA ± ; CD43 ± ; CD30+; MUM-1+; IRF4+; CD5−; CD2−; CD3−; CD4−; CD8−; HHV8+; podoplanin−; CD34−; CD31−; S100−; CD138−; CD79 alpha−; CD20−; CD68±; CD56−; CD15−; MPO focal positivity; EBNA negative—Fig. 2. Clinical data and histological results were interpreted as HHV8-related extranodal PEL. A bone marrow biopsy was performed and resulted completely negative. After 6 weeks of ART, progressive disappearance of the masses was noted, and the control PET-CT scan resulted completely negative at 3 months. After 19 months of ART the patient was in complete remission of extranodal PEL; HIV viremia was undetectable (< 20 copies/mL), CD4 count was 766 cells/mL (26%; CD4/CD8 ratio 0.55) and HHV8 viremia was undetectable.

## Discussion and conclusion

The role of HHV8 as the causative agent of PEL was first reported in 1995; its DNA was found in all the analysed AIDS-related body-cavity-based lymphoma fluids in serous cavities and was found to be involved in the classic form of PEL [5]. In 2004, a solid, or extracavitary (EC-PEL), variant of PEL was described and defined by the presence of solid tumour masses in different organs with or without serous cavity involvement. Both variants have been formally recognized by the WHO in the 2008 classification of lymphoid neoplasm [7, 25]. The spectra of haematologic malignancies related to HHV8 infection has broadened since its discovery. HHV8-associated lymphoid proliferations are uncommon disorders, they encompass different diseases with overlapping clinical and immunohistochemical features that complicates their classification. Furthermore, there is a significant number of case reports describing HHV8-associated lymphoid alterations difficult to classify following the latest WHO criteria, expanding the spectrum of viral-associated lymphomas. Anaplastic large cell lymphoma (ALCL), diffuse large B cell lymphoma NOS (DLBCL-NOS) and germinotropic lymphoproliferative disorder (GLPD), a localized lymphadenopathy that typically affects immunocompetent patients and has a favourable response to chemotherapy, are some of the haematological malignancies included in the differential diagnosis of EC-PEL [26, 27]. Morphologic analysis of PEL showed some common features with diffuse large B cell lymphoma and ALCL, but the immunophenotypic and genetic characteristics indicate that PEL/EC-PELs cells derive from terminally differentiated B lymphocytes that have gone through the germinal centre processes, while ALCL and DLBCL derive from naïve B cells [28–30]. GLPD derives from a germinal centre B cell but is polyclonal, mainly occurs in immunocompetent patients, involves only lymphatic stations and is characteristically Epstein Barr Virus (EBV) related [7, 30]. PEL and extracavitary PEL differ in the clinical presentation, while they have similar morphologic, immunophenotypic and genetic features [25, 31]. PEL is characterized by a “null-cell” phenotype, typically lacking pan B cell antigens (CD19, CD20, CD79a), express markers of terminal B cell differentiation such as MUM1 and CD138 (the latter frequently absent), and CD30 is usually positive. EC-PEL cells share the same immunophenotypic features, but they express B cell associated antigens slightly more often, and more frequently express aberrant T cell markers [7, 30].

The detection of HHV8 presence in neoplastic cells is needed for the definitive diagnosis of PEL and is usually demonstrated through the detection of HHV8 viral proteins (LANA-1); 80% of tumour cells are also co-infected with EBV.

There is a wide variety of uncommon lymphoproliferative disorders correlated with HHV8 infection, with significant morphologic and phenotypic overlapping features, complicating their classification and representing a diagnostic challenge. In the described case extracavitary PEL diagnosis was guided both by clinical and immunohistochemical features: he is a young man, HIV positive ART naïve with a low CD4 count, HHV8 high viremia and multiple subcutaneous masses with cells that show a null phenotype and are CD30 + and HHV8 positive.

PEL/EC-PEL are characterized by a very poor prognosis, with an overall survival (OS) of about 6 months even with aggressive chemotherapy, which is considered the first line treatment. Poor prognosis factors are represented by the absence of antiretroviral therapy prior to diagnosis, poor performance status, the number of cavities involved and a high HHV8 viremia with > 40,000 copies/mL [26, 32–35]. A recent retrospective analysis from the American National Cancer Database demonstrated different survival rates based on the primary site of PEL, with the longest OS being for intrathoracic origin, followed by extracavitary and peritoneal localizations [36]. The prognostic value of PEL localization is not univocal, with controversial data existing from small groups of patients [8]. The increase of CD4 cell count and the immune restoration have been suggested to play a role in controlling HHV8 replication and HHV8-related tumours progression [9, 37, 38].

Given the rarity of PEL no standard of care exists for the treatment. A combination therapy with a cyclophosphamide, doxorubicin, vincristine and prednisone (CHOP)-based regimen and ART are commonly used as first line therapy [8, 9]. Case reports where bortezomib, intracavitary cidofovir, valganciclovir and monoclonal antibodies were used as synergic drugs for PEL treatment have been published, showing controversial results [10–19]. The ability of valganciclovir to reduce HHV8 replication has been studied, and have shown efficacy, but no randomized clinical trials have been conducted to assess the therapeutic impact of the drug in HHV8-related diseases and there is no consensus on whether antiviral therapy might be clinically useful in controlling HHV8 replication and tumour proliferation [39–41]. Recent case reports described a longer OS free of disease in patients treated with ART, chemotherapy and ganciclovir administered first intravenously then orally [13, 20, 21]. In the reported case, the use of ganciclovir might have played a role in controlling the haematological disease.

A fast recovery of the immune status is correlated with a better prognosis, so the rapid initiation of ART is essential [9]. It has been demonstrated that using ART with protease inhibitors or non-nucleoside transcriptase inhibitors could reduce HHV8-related tumours [42–46], while little and contradictory information exists regarding integrase inhibitors (INI) [47].

In the reported case, the patient had several factors indicating a poor prognosis, the patient was HIV-treatment naive and presented with an HHV8 viremia > 40,000 copies. Prompt initiation of ART with TAF/FTC and DTG led to a rapid immune restoration with subsequent PEL regression. Most likely, the concomitant intravenous ganciclovir treatment for CMV chorioretinitis contributed in the HHV8-related malignancy control. In 1998, regression of a case of classic PEL after ART initiation was described and to our knowledge, what we present here is the first reported case of an extracavitary PEL successfully treated with ART alone, without using chemotherapy [37].

HHV8 has been associated with different variety of tumours and lymphoproliferative disorders. The oncogenic mechanism of HHV8 infection relate to its ability to inhibit tumour suppressor genes, impair apoptosis and promote cell proliferation and systemic inflammation trough the release of viral oncogenic products, cytokines and growth factors that are human analogues, such as LANA-1, viral (v) cyclin, vInterleukin (vIL)-6 and vIL-8 [3, 19, 32, 48, 49]. Novel therapeutic strategies are focusing on the inhibition of the inflammatory and apoptotic pathways activated by the virus, with different results [1, 19, 50–54]. Ganciclovir, and other antiviral drugs such as cidofovir and foscavir, have a direct inhibitory activity on HHV8 replication, and have been used in HHV8-related tumours, often in association with chemotherapy. In HIV-infected patients, a prompt ART initiation followed by the immune restoration has been associated with a decrease in HHV8 replication and a better outcome in HHV8 related malignancies, as documented in HIV-infected patients with KS [42–45]. Whether to try or not a conservative approach in HIV-infected PEL patients, must be carefully evaluated, considering the patient’s characteristics and the prognostic factors.

## Data Availability

Not applicable. Data sharing is not applicable to this article as no dataset were generated or analysed during the current study. Authors can confirm that all the relevant data are included in the article and/or its additional files.

## Abbreviations

Ab: antibodies
AIDS: acquired immunodeficiency syndrome
ALCL: anaplastic large cell lymphoma
ART: antiretroviral therapy
CHOP: cyclophosphamide, doxorubicin, vincristine and prednisone
CMV: cytomegalovirus
CRP: C reactive protein
CT: computed tomography
DLBCL-NOS: diffuse large B cell lymphoma-not otherwise specified
DNA: deoxyribonucleic acid
DTG: dolutegravir
EA: early antigen
EBNA: Epstein–Barr Virus Nuclear Antigen
EBV: Epstein Barr Virus
EC-PEL: extracavitary primary effusion lymphoma
GLPD: germinotropic lymphoproliferative disorder
HAV: Hepatitis A virus
Hb: haemoglobin
HBsAb: hepatitis B surface antibodies
HBV: hepatitis B virus
HCV: hepatitis C virus
H&E: Haematoxylin–eosin
HIV: human immunodeficiency virus
HHV8: human herpes virus 8
HSV1: herpes simplex virus 1
HSV2: herpes simplex virus 2
IgG: immunoglobulin G
IgM: immunoglobulin M
IL: interleukin
INI: integrase inhibitors
IRF4: interferon regulatory factor 4
KS: Kaposi’s sarcoma
KSHV: Kaposi’s sarcoma-associated herpesvirus
LANA-1: latency-associated nuclear antigen
LCA: leukocyte common antigen
MCD: multicentric Castleman disease
MPO: myeloperoxidase
MUM-1: multiple myeloma 1
NA: not applicable
NHL: non Hodgkin lymphoma
OS: overall survival
PEL: primary effusion lymphoma
PET-CT: positron emission tomography-computed tomography
TAF/FCT: tenofovir alafenamide/emtricitabine
TB: tuberculosis
v: viral
VCA: viral capsid antigen
WBC: white blood cells
WHO: World Health Organization
